# Transcriptomics Analysis of Testis Development in *Thamnaconus septentrionalis* Responding to a Rise in Temperature

**DOI:** 10.3390/ani16020327

**Published:** 2026-01-21

**Authors:** Yan Liu, Xueli Zhang, Wengang Xu, Jiulong Wang, Li Bian, Yanqing Wu, Meng Li, Liming Liu

**Affiliations:** 1 School of Ocean, Yantai University, Yantai 264005, China; 2 State Key Laboratory of Mariculture Biobreeding and Sustainable Goods, Yellow Sea Fisheries Research Institute, Chinese Academy of Fishery Sciences, Qingdao 266071, China; 3 East China Sea Fisheries Research Institute, Chinese Academy of Fishery Sciences, Shanghai 200090, China; 4 Laboratory of Marine Ecology and Environmental Sciences, Institute of Oceanology, Chinese Academy of Sciences, Qingdao 266071, China

**Keywords:** gonad development, molecular regulation, transcriptomics, *Thamnaconus septentrionalis*

## Abstract

*Thamnaconus septentrionalis* is an emerging commercially important aquaculture species in China, distributed extensively in the Indo-West Pacific Ocean. Recently, because of the seriously declining population and considerable economic potential of this aquaculture fish, increasing attention has been paid to the conservation and development of *T. septentrionalis*. Artificial fish breeding is essential and has become progressively implemented on local farms in China, which benefits the protection of *T. septentrionalis* resources and facilitates the development of its fishery industry. Previous studies have demonstrated that temperature could significantly influence ovary development in *T. septentrionalis*. However, the potential molecular mechanisms underlying the influence of temperature on testis development in *T. septentrionalis* have been scarcely studied. Thus, this study comprehensively explores the effects of temperature on testis development in *T. septentrionalis* using histological observation and transcriptomic techniques. Histological and transmission electron microscopy analyses indicated that *T. septentrionalis* testes, undergoing a rise in temperature, developed from phase III to IV. Transcriptomic analysis identified 315 differentially expressed genes, including 200 upregulated and 115 downregulated genes. Moreover, rising temperatures may enhance testis development by regulating steroid hormone biosynthesis, cellular senescence, and nucleotide metabolism. The upregulation of four genes (*hsd11b2*, *cyp11b*, *cyp11a*, and *hsd17b3*) involved in the steroid hormone biosynthesis process may significantly contribute to the increased level of testosterone and 11-keto-testosterone. This study is the first to elucidate the potential molecular mechanism involved in *T. septentrionalis* testis development induced by temperature, offering valuable and novel insights for its artificial breeding and fishery resources conservation.

## Simple Summary

Fish are rich in species and provide an important food supply, especially high-quality protein sources, for billions of people worldwide. In recent years, aquaculture production of fish has exceeded wild catch production, highlighting the importance of the fish aquaculture industry. As is well-known, regulating the reproductive processes of economically valuable fish is a critical step in artificial breeding. The gonadal development of many fish species, including the greenfin horse-faced filefish in this study, could be influenced by temperature. Thus, the present study first investigated the potential molecular mechanism of *Thamnaconus septentrionalis* testis development induced by temperature and found that the rising temperature enhanced its testis development. The major findings will provide a valuable and important theoretical and technical basis for the artificial breeding and fishery resources conservation of *T. septentrionalis* in the future.

## 1. Introduction

*Thamnaconus septentrionalis* is extensively distributed in the coastal lines around the Chinese, Japanese, and Korean Peninsula regions. It is a significant commercial fish species with characteristic spine-like first dorsal fins and blue-green fins [1]. Since the 1980s, overfishing and ruining the environment have resulted in a sharp decrease in *T. septentrionalis* populations, which are insufficient to meet market requirements [2]. In 2023, the marine catch production of filefish reached 122,258 tons, as reported in the China Fisheries Statistical Yearbook [3]. Artificial breeding is essential for developing the *T. septentrionalis* fishery industry and meeting market needs, with seedling generation expanding rapidly alongside aquaculture development.

During *T. septentrionalis* breeding, we observed that the ovaries and testes of the female and male individuals were maintained at stages II and III, respectively, without appropriate temperature stimulation. In the natural and cultural environment, from May to June along the Shandong coastal waters, the gonads of parent fish can initiate further development with rising temperatures. The fry cultivated under normal temperatures can hardly be marketed in the same year. In November–December, we increased the temperature of culturing parent fish to accelerate gonadal development as well as maturation of *T. septentrionalis* during the artificial breeding period [4]. The molecular mechanism involved in promoting gonadal development and maturation in *T. septentrionalis* with increasing temperature remains unclear.

In teleosts, gonadal development is a crucial factor for fish reproduction and population sustainability, which is influenced by numerous environmental factors, with water temperature playing an essential role in this process [5,6]. Research has revealed that suitable higher temperatures can contribute to gonadal development [7,8]. However, most research mainly focused on the relationship between ovary development and temperature [9,10,11,12], and recently, the transcriptomic technique has been employed for systematic investigation in the regulatory mechanisms of ovary development in the common carp [13]. In addition, a limited number of previous studies indicated that the testis developmental process of Atlantic salmon and small yellow croaker could be influenced by temperature [14,15]. However, the molecular mechanism of promoting testis development and maturation in the economically important fish *T. septentrionalis* by rising temperatures remains unexplored. Thus, in this study, we applied the transcriptomics technique to systematically elucidate the molecular mechanisms of testis development in *T. septentrionalis* induced by temperature rise. Moreover, histological and ultrastructural observations of the *T. septentrionalis* testis were performed. The concentrations of three important steroid hormones (17 β-estradiol; E2, 11-keto-testosterone; 11-KT, and testosterone; T) were detected in the *T. septentrionalis* plasma in this study.

## 2. Materials and Methods

### 2.1. Ethical Statement

All of the experiment procedures related to animals received approval from the Animal Care and Use Committee of Yantai University, China (Permit Number 20170605).

### 2.2. Experimental Animals, Design, and Sampling

A total of 12 male *Thamnaconus septentrionalis* (3 years old) were acquired from an aquatic hatchery farm in Yantai City, China. During reproductive control by temperature, testes of *T. septentrionalis* (n = 6 per group) were obtained before water warming and set as the low temperature (LT) group treated with 13.2 °C, whereas the other six individuals collected after water warming were set as the high temperature (HT) group treated with 14.2 °C. The information on experiment design in the present study is shown in Figure S1. Before sampling, the experimental fish were not fed for one day and were treated with MS-222 (0.05%). The indices of fish body weight, full-length, and gonad weight were carefully calculated and recorded (Table 1). Blood samples were extracted from *T. septentrionalis* with a heparinized syringe. Fish blood was extracted from the caudal vein of 12 individuals (n = 6 for each group) and carefully centrifuged at 800× *g* for 15 min to obtain the plasma for further measurement of hormones. Testes tissues were sampled for the subsequent molecular and cellular analyses. For the testis sample, about 0.5 cm^3^ of testis was kept in the Bouin’s solution for histopathology, and 0.5 cm^3^ of testis tissue was preserved in the solution (glutaraldehyde 2.5%) for transmission electron microscope (TEM) analysis. The other testes tissues of *T. septentrionalis* (n = 3/group for transcriptomic analysis) were collected to be preserved at −80 °C after quick freezing in liquid nitrogen until further processing.

### 2.3. Assays of the Gonadal Somatic Index and Plasma Hormones Analysis

The gonadal somatic index (GSI) of each sampled fish was carefully measured and recorded, according to the previous study [16]. The concentration levels of the three steroid hormones (E2, T, and 11-KT) were examined by the commercial ELISA kit (Mlbio, Shanghai, China).

### 2.4. Histopathology of T. septentrionalis Testes

*Thamnaconus septentrionalis* testes (n = 6 for each group) were dissected for further histological assay. After being excised and fixed in 4% formaldehyde for 24–48 h, the testes tissues were kept in 70% ethanol. All tissue samples were subjected to paraffin histology with the hematoxylin and eosin (H&E) staining method [17]. The slides were observed and photographed carefully using an Olympus BX53 microscope (200× and 400×) (Olympus Corporation, Tokyo, Japan).

### 2.5. Ultrastructural Observation of T. septentrionalis Testes

A 0.1 M sodium cacodylate buffer (pH 7.4) enriched with 2.5% glutaraldehyde was utilized to fix the testis tissues at 4 °C for 24 h, then three washes were conducted (5 min each) with the same buffer, and a 1.5 h post-fixation was conducted in 1% osmium tetroxide. Samples underwent dehydration in a graded acetone series, embedding in Epon 812, sectioning with a microtome, staining with uranyl acetate and lead citrate, and a HITACHI HT7800 electron microscope (Hitachi High-Tech Corporation, Tokyo, Japan) was utilized for examination.

### 2.6. Total RNA Extraction, Library Construction, Transcriptomics Analysis

Beijing Novogene Technology Co., Ltd. (Beijing, China) was employed to conduct the total RNA extraction of the collected *T. septentrionalis* testes tissues (n = 3 for each group), library preparation, and subsequent transcriptome sequencing and bioinformatics analyses. The bioinformatics analysis was conducted according to the previous study [18].

### 2.7. qRT-PCR Validation of Representative DEGs

From the transcriptome sequencing data, we chose 16 DEGs related to testis development to detect their gene expressions by qRT-PCR analysis to further validate the reliability of transcriptomic data. The Gene ID, amplicon size, primer efficiency, and annealing temperature of the 16 DEGs are presented in Table S1. Total RNA isolation was conducted from the same testis samples via TRIzol reagent and exposed to RNase-free DNase I (TaKaRa, Japan) to eliminate genomic DNA. The PrimeScript™ RT reagent Kit (TaKaRa, Japan) was utilized to conduct the cDNA synthesis. The qRT-PCR reaction using the primer concentration of 10 μM was conducted according to Li et al. [19]. The qRT-PCR efficiency was assessed from the given slope in Rotor-gene Real-Time PCR System Manager software (version 2.3.1) by a 10-fold diluted cDNA sample series with six dilution points [19]. Efficiency (*E*) was determined by the equation *E* = 10 ^(−1/slope)^ [20]. qRT-PCR was carried out in triplicate and used the 2^−∆∆CT^ relative quantification method to quantify the gene expression levels [21]. Transcript level normalization to the geometric mean of 3 reference genes (elongation factor 1-α, GAPDH, and β-actin) was conducted according to the MIQE guidelines [22] to guarantee the accuracy and reliability of the gene expression levels. Primers (Table S1) were designed using qPrimerDB v2.0 [23]. Data are reported as mean ± standard deviation (SD), and the independent samples *t*-test was utilized to conduct statistical analyses in GraphPad Prism 8 (USA), with significance set at *p* < 0.05.

## 3. Results

### 3.1. Physiological Indexes of the Experimental Male T. septentrionalis

No significance was detected in the total length, testis weight, or body weight between the LT and HT groups. However, the GSI was found to be increased significantly in the HT group (1.71-fold, *p* < 0.05) compared to the LT group (Table 1).

### 3.2. Histology and TEM Assays of T. septentrionalis Testis

**Figure 1:**
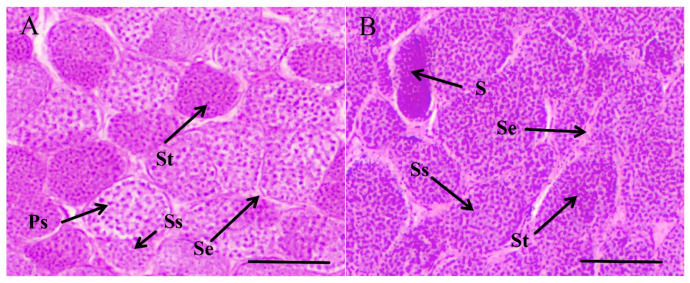
Histology of *T. septentrionalis* testes in LT (**A**) and HT (**B**) groups. Ps, primary spermatocytes; Ss, secondary spermatocytes; Se, Sertoli cell; St, spermatid; S, spermatozoa. Scale bar: 100 μm.

Histological observations indicated that LT testes were enriched with a significant number of the primary spermatocytes (Ps), spermatids (St), and secondary spermatocytes (Ss), indicating that LT testes were at stage III. The Ss were observed to be comparatively smaller than the Ps. Non-significant changes were found in the Sertoli cell (Se) between the LT and HT groups. Then, as the temperature increased, the testes started to develop in the presence of a limited quantity of spermatids (St) and spermatozoa (S) (Figure 1).

**Figure 2:**
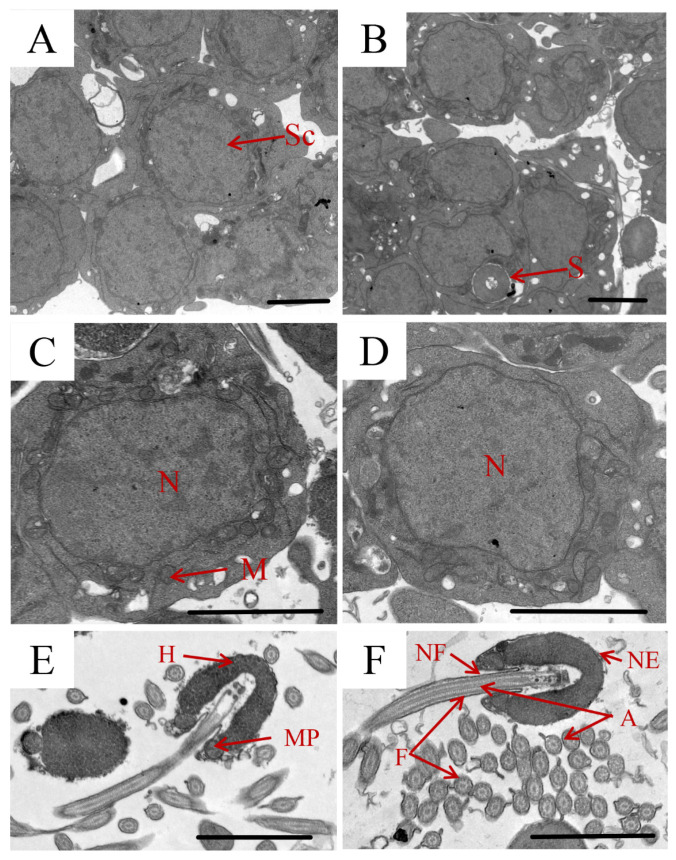
TEM analysis of *T. septentrionalis* testes in LT (**A**,**C**) and HT (**B**,**D**) groups. TEM analysis of the *T. septentrionalis* spermatozoa in LT (**E**) and HT (**F**). Sc, spermatocytes; S, sperm; N, nucleus; NF, nuclear fossa; MP, mid-piece; NE, nuclear envelope; F, flagellum; M, mitochondrion; A, axoneme; H, head. Scale bar: 1 μm.

By the TEM analysis, the only spermatocytes of *T. septentrionalis* were found in the testes of the LT group (Figure 2A), while the presence of sperm was observed obviously in the testes of the HT group (Figure 2B), which was consistent with the histological findings. Spermatocytes exhibited oval-shaped nuclei with regular outlines, and the size of the nuclei was ~3.5 μm in diameter. And certain mitochondria were distributed in the cytoplasm around the nuclei (Figure 2C,D). In addition, the nucleus in the LT group (Figure 2C) seemed to be a little stretched and more condensed than that in the HT group (Figure 2D). The sperm structure consisted of three dominant parts, including a head without acrosomes, a mid-piece, and a single flagellum. The flagellum axoneme comprised microtubule configurations in a ‘9 + 2’ microtubule pattern (Figure 2E,F). In addition, the cellular structure of *T. septentrionalis* sperm was found to be better developed in the HT group (Figure 2F) than in the LT group (Figure 2E).

### 3.3. E2, T, and 11-KT Levels of T. septentrionalis Testes in Response to Temperature Rise

**Figure 3:**
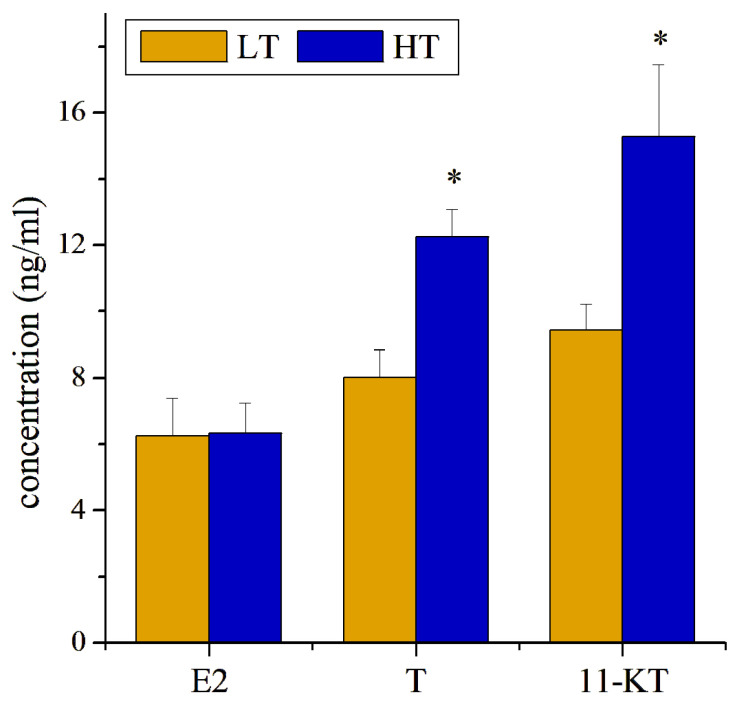
The concentration of the plasma 17β-estradiol (E2), testosterone (T), and 11-ketotestosterone (11-KT) in LT and HT groups. * on bars represent statistical significance (*p* < 0.05).

The serum levels of E2, T, and 11-KT in *T. septentrionalis* were measured and are shown in Figure 3. In comparison with the LT group, the concentration levels of T (1.52-fold change, *p* < 0.05) and 11-KT (1.62-fold change, *p* < 0.05) were elevated with significance in the fish plasma in the HT group, whereas that of E2 exhibited no significant difference (Figure 3).

### 3.4. Transcriptome Profiles of T. septentrionalis Testes Responding to Temperature

Transcriptomic analysis of *T. septentrionalis* testes yielded 40.95 Gb of raw bases corresponding to the LT (21.0 Gb) and HT (19.95 Gb) groups, with 39.39 Gb clean bases obtained in the LT (20.15 Gb) and HT (19.24 Gb) groups after eliminating low-quality and junction reads. In the LT and HT groups, the average GC contents were 52.17% and 52.34%, average Q30 values 91.09% and 91.02%, and average Q20 values 96.37% and 96.34%, respectively. Clean reads aligned to the genome of *T. septentrionalis* with mean rates of 78.14% (LT) and 78.27% (HT), confirming the high quality and reliability of the sequencing data (Table S2).

**Figure 4:**
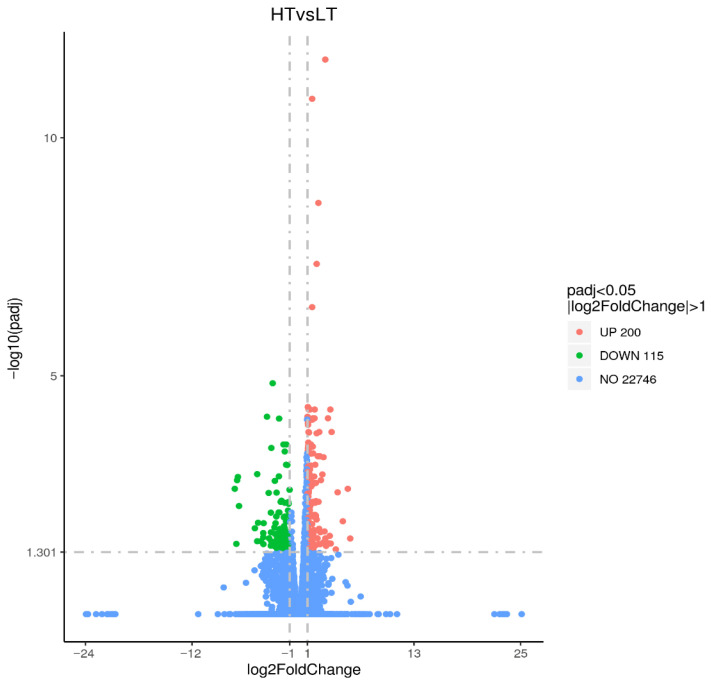
The differentially expressed genes (DEGs) between the LT and HT groups. Red dots indicated significantly upregulated DEGs, green dots indicated significantly downregulated DEGs, and blue dots indicated non-significantly expressed genes.

**Figure 5:**
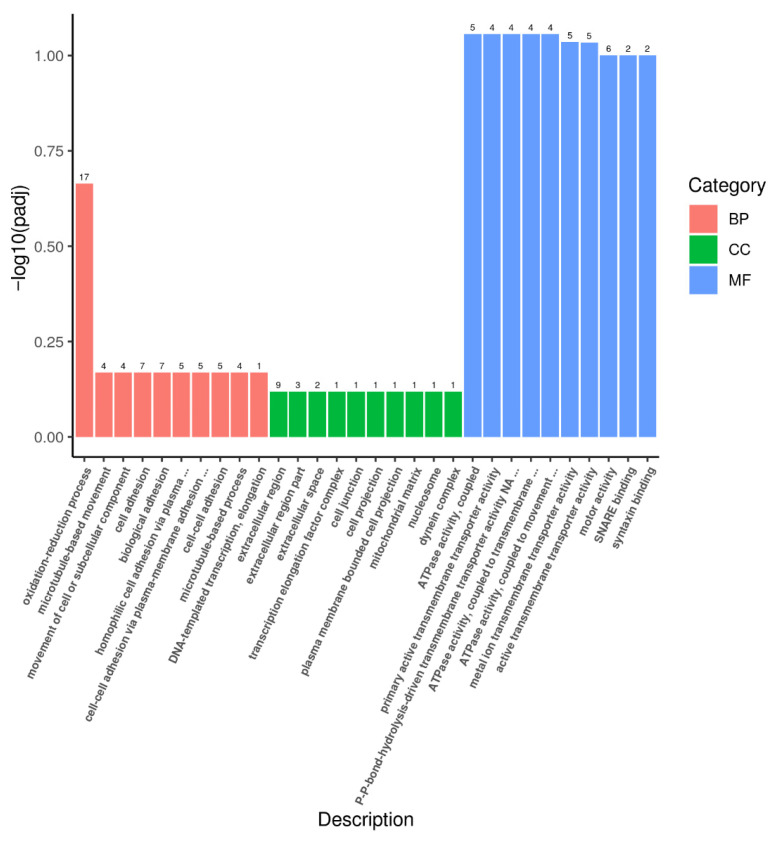
GO enrichment analysis of DEGs between the LT and HT groups. The numbers on each bar represent the number of differentially expressed genes identified in the corresponding GO term. Biological process, BP; molecular function, MF; and cellular component, CC.

**Figure 6:**
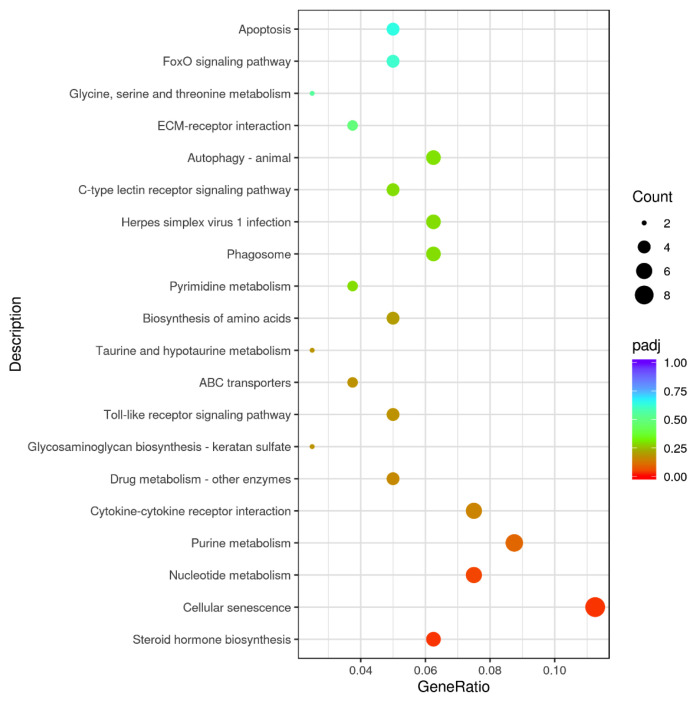
KEGG pathway analysis of DEGs between the LT and HT groups.

By comparative analysis of the transcriptomics data of LT and HT groups with the screening circumstances of | log_2_ FC | ≥ 1 and FDR < 0.05, we identified 315 DEGs, consisting of 200 upregulated and 115 downregulated genes (Figure 4). To further investigate the biological processes involved in the DEGs, the GO analysis was performed. The findings indicated that DEGs were primarily classified into biological processes (BP), molecular functions (MF), and cellular components (CC). The biological processes primarily comprise oxidation–reduction, microtubule-based movement, movement of cell or subcellular components, cell adhesion, and biological adhesion. The cellular constituents primarily include the extracellular region. Molecular functions were mainly relevant to SNARE binding, syntaxin binding, ATPase, transmembrane transporter, motor, oxidoreductase, and monooxygenase activities (Figure 5). KEGG analysis illustrated that 315 DEGs were significantly enriched in multiple pathways, including steroid hormone biosynthesis, cellular senescence, nucleotide metabolism, purine metabolism, cytokine–cytokine receptor interaction, toll-like receptor, ABC transporters, and biosynthesis of amino acids (Figure 6).

### 3.5. qRT-PCR Analysis of Critical DEGs in T. septentrionalis Testis

**Figure 7:**
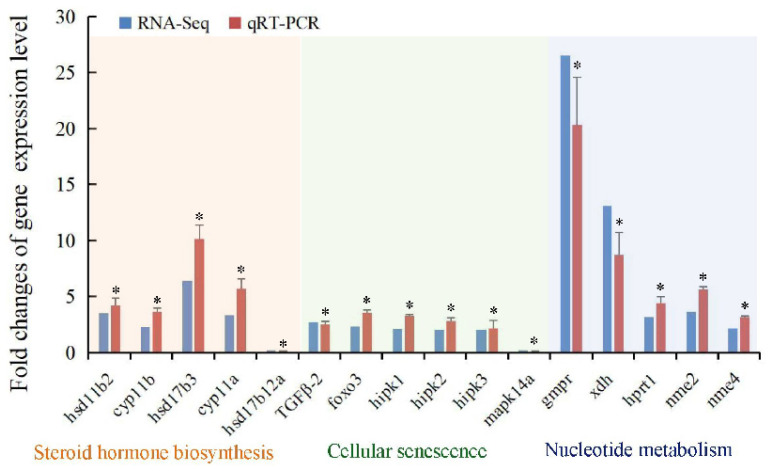
Comparison of qRT-PCR and RNA-Seq results for transcripts of 16 DEGs in *T. septentrionalis* testes treated with temperature. The fold changes of the gene expressions (HT/LT) obtained by the transcriptomic data and qRT-PCR validation analysis are shown in the blue and red bars, respectively. The statistical significance of the fold-change between HT and LT, validated by qRT-PCR analysis in red bars, is indicated by an asterisk (*) (*p* < 0.05).

To verify the accuracy and reliability of the transcriptomics data, a total of 16 DEGs in *T. septentrionalis* testes involved in the pathways of steroid hormone biosynthesis (*hsd11b2*, *cyp11b*, *cyp11a*, *hsd17b3*, and *hsd17b12a*), cellular senescence (*TGFβ-2*, *foxo3*, *hipk1*, *hipk2*, *hipk3*, and *mapk14a*), and nucleotide metabolism (*gmpr*, *xdh*, *hprt1*, *nme2*, and *nme4*) were subjected to qRT-PCR analysis. qRT-PCR findings indicated that the DEGs showed a similar expression trend comparable with that of the sequencing data (Figure 7), reflecting the accuracy and reliability of the transcriptomics data. In addition, fourteen of the examined DEGs were significantly upregulated in the HT group compared to the LT group (*p* < 0.05), except for the significant downregulation of *hsd17b12a* and *mapk14a* in the HT group compared to the LT group (*p* < 0.05). The similar trends of the gene expression changes between the transcriptomic and qRT-PCR analyses (Figure 7) reflected the accuracy and reliability of the transcriptomic data in the present study.

## 4. Discussion

*Thamnaconus septentrionalis* is a commercially important marine fish broadly spread in the Indo-West Pacific and a key aquaculture species in China [1]. Recently, *T. septentrionalis* has been confronted with a serious decrease in its population resources and the problem of overfishing [1]. Previous studies have examined its reproductive biology and artificial propagation under aquaculture conditions [4,24,25]; the molecular mechanisms regulating reproductive development remain unclear. In this study, we attempted to investigate the molecular regulation mechanism of *T. septentrionalis* testis development induced by temperature. The critical genes as well as biological processes associated with the temperature-induced testes development of *T. septentrionalis* were uncovered. The significantly high gonadal somatic index (GSI) of the HT group in this study reflected that temperature rise likely promoted the testis development of *Thamnaconus septentrionalis*.

The developmental processes of fish gonads could be influenced by both genetic and environmental factors, especially sensitive to temperature [8,26]. Housh et al. [27] found that elevated temperature significantly influenced the ovarian development of the pupfish via regulation of the reproductive endocrine pathways. Zhao et al. [28] suggested that the high-temperature treatment could result in the female sex reversal of the Nile tilapia *Oreochromis niloticus*. Wright et al. [29] suggested that the temperature rise (+5 °C) delayed ovarian development in the lesser sandeel *Ammodytes marinus*, with the timing of oocyte maturation over two months. Spinks et al. [30] found that an elevated temperature (+1.5 °C) consistent with the projected ocean warming could increase the breeding probability in the females, while male development or pairs reproducing was affected negatively by higher temperature in the coral reef damselfish *Acanthochromis polyacanthus*. The present study suggested that the elevated temperature (+1 °C) contributed to testis development in cultured *T. septentrionalis.* The different effects of temperature rise on male development between Spinks et al. [30] and this study were likely due to the different experimental treatments and species. In addition, given that the occurrence of fish reproduction was typically within a narrow thermal window, our results implied that an increase of +1 °C might facilitate the optimal window for reproduction in male *T. septentrionalis*.

Further transcriptomic analysis indicated that the steroid hormone biosynthesis process was modulated with significance in *T. septentrionalis* testes with increasing temperature. Biosynthesis of steroid hormones, undergoing three classes of sex steroids (androgens, estrogens, and glucocorticoids), was initiated from a common precursor molecule (cholesterol) and a series of reactions catalyzed by cytochrome P450 hydroxylases and hydroxysteroid dehydrogenases in mitochondria [31,32,33,34,35]. It plays a very important regulatory role in developing the reproductive system and maintenance of testicular function [33,36,37]. In this study, the transcripts of four genes (*hsd11b2*, *cyp11b*, *cyp11a*, and *hsd17b3*) involved in the steroid hormone biosynthesis process were significantly enhanced in *T. septentrionalis* testes after the temperature rise. CYP11A is a mitochondrial monooxygenase, catalyzing the conversion of cholesterol to pregnenolone and participating in the first step in steroid biosynthesis [38]. HSD17B3 plays a key role in male sex development by converting androstenedione to the primary male sex steroid hormone T. CYP11B1 and HSD11B2 are crucial in 11-KT production in the testis [39,40]. The upregulation and activation of these genes might be an important reason for the observed increase in T and 11-KT hormones in this study. Li et al. [41] also found an elevating trend of plasma 11-KT concentration during seahorse testicular development. Moreover, T and 11-KT have been demonstrated to be potent in androgen-mediating spermatogenesis, sexual characteristics, and male fertility in teleosts [42,43]. Treatment with androgen inhibitors or androgen mutations blocks spermatogenesis, with fewer spermatocytes and sperm observed in the zebrafish testes [44]. In this study, the number of sperm was significantly increased, and the testis developed to stage IV with increasing temperature. These findings highlighted the critical role of steroid hormone biosynthesis in testis development and spermatogenesis in *T. septentrionalis* in response to rising temperature.

The cellular senescence pathway was also affected remarkably in *T. septentrionalis* testes after the temperature rise treatment. Cellular senescence, a stress response condition in the context of homeostasis, promotes tissue remodeling through serial related genes [45]. In this study, except for the significant downregulation of *mapk14a*, the mRNA transcripts of five genes (*tgfβ-2*, *foxo3*, *hipk1*, *hipk2*, *and hipk3*) involved in the cellular senescence pathway were increased significantly in *T. septentrionalis* testes responding to temperature rise. Transforming growth factor beta participated in maintaining effective peripheral immune tolerance in sperm and normal sperm development [46,47]. FOXO3 could inhibit the formation of StAR proteins to affect testosterone synthesis in mice [48]. Ni et al. [49] demonstrated that treatment with nicotinamide riboside or metformin resulted in inhibition of apoptosis in Basonuclin 1 (Bnc1)-knockdown spermatogonia by stimulating CREB/SIRT1/FOXO3 signaling pathway. HIPKs play conserved roles in the initiation of spermatogenesis and sperm maturation [50]. In addition, Dong et al. [51] found the upregulation of *hipk1/2/3* and *foxm* in four-eyed sleepers and suggested its important role in spermatogonia differentiation and sperm production in comparison to the testis at 3 months post hatch (mph). In addition, mitogen-activated protein kinases (MAPKs) are evolutionarily conserved proteins and found to be crucially involved in testis development by regulating spermatogenesis [52,53,54]. Thus, the significant downregulation of *mapk14a* in this study might also be associated with the testis development in *Thamnaconus septentrionalis*. Collectively, the significantly enriched pathway “cellular senescence pathways” participated in regulating testis development and gametogenesis in *T. septentrionalis*.

The nucleotide metabolic pathway was also influenced significantly in *T. septentrionalis* testes after the temperature rise treatment. Qi et al. [55] discovered that glyphosate-based herbicides induce reproductive toxicity by impairing energy and nucleotide metabolism in mouse testes, suggesting that nucleotide metabolism is essential for maintaining normal testis development. In the present study, five genes (*gmpr*, *xdh*, *hprt1*, *nme2*, and *nme4*) involved importantly in these pathways were significantly enhanced in *T. septentrionalis* testes responding to temperature increase. Guanosine monophosphate reductase (GMPR) plays an important role in maintaining the intracellular balance of adenine and guanine nucleotides by converting guanosine nucleotides to pivotal precursors of both G and A nucleotide [56]. Xanthine dehydrogenase (XDH) could reduce NAD+ to NADH. Previous studies have suggested that an increased NADH/NAD+ ratio can affect testis development [49,57,58]. Hypoxanthine phosphoribosyl transferase 1 (HPRT1), an enzyme in the DNA salvage pathway, participates in transferring phosphoribose from phosphoribosyl diphosphate to hypoxanthine and guanine bases, which are involved in the regulation of purine synthesis in the cell cycle [59]. NME2 and NME4 belong to the conserved NME protein family and play critical roles in maintaining cellular nucleoside triphosphate homeostasis [60,61]. Therefore, in principle, HPRT1, NME2, and NME4 activities could control the biological processes (e.g., DNA synthesis or GTP-dependent biological activities) and mediate the basic cellular functions (e.g., differentiation and development). So, the present study suggested that the significantly enriched pathway “nucleotide metabolism pathway” likely plays a key role in maintaining normal testis development in *T. septentrionalis*.

## 5. Conclusions

This study comprehensively examined the potential underlying molecular mechanism of testis development in *T. septentrionalis* induced by the temperature rise at both the molecular and cellular levels. The present study suggested that temperature rise could promote the *T. septentrionalis* testis development. Critical genes and important regulatory pathways involved in *T. septentrionalis* testis development were revealed. The major findings of this study may support the seedling production of this fish species and enhance our understanding of the theoretical evidence of temperature-induced testis development in artificial seed production of this important, economically valuable fish species.

## Figures and Tables

**Table 1:** Physiological indices of *Thamnaconus septentrionalis* in this study.

Group	Total Length (cm)	Body Weight (g)	Testis Weight (g)	GSI (%)
LT	28.40 ± 3.99	385.86 ± 111.44	1.87 ± 0.73	0.48 ± 0.07
HT	29.67 ± 2.58	406.43 ± 125.03	3.60 ± 1.77	0.82 ± 0.23 *

Note: * on GSI indicate statistical significance (*p* < 0.05).

## Data Availability

All data are available in Supplementary Data Files. The RNA-Seq data for the transcriptome analysis are available via the BioProject accession number PRJNA1227174.

## Supplementary Materials

The following supporting information can be downloaded at https://www.mdpi.com/article/10.3390/ani16020327/s1. Table S1: Primers used for qRT-PCR assays in this study; Table S2: The statistics of the transcriptomic data in this study; Figure S1: The information of the experimental design in this study.
