# Supplementary material for: Transcriptomics Analysis of Testis Development in Thamnaconus septentrionalis Responding to a Rise in Temperature
Source: Animals (Basel). 2026 Jan 21;16(2):327. doi: 10.3390/ani16020327 (PMC12837544; doi:10.3390/ani16020327)
Supplement: Supplementary file 1 [file animals-16-00327-s001.zip › animals-4077258-supplementary/Table S1.pdf]

Table S1 The qRT-PCR primers used in this study.

Genes	Gene ID	Forward primer sequence (5'-3')	Reserve primer sequence (5'-3')	Amplicon size (bp)	Primer efficiency	Annealing temperature (°C)
hsd11b2	EVM0021207.1	TGCAGGAGGAAGTGGGTATAGA	ATCCTTGTGTGAGGCCATCATT	236	96.4%	60
cyp11b	EVM0020876.1	ATCAGCTGACGTTCAAGGATGT	GCTGAAGGTCTTCTCCATGTGA	280	98.5%	60
hsd17b3	EVM0017052.1	CGGTGTTTAAAGCACGCACA	CGTTCTGTCAGCCCACTCAT	266	94.9%	60
cyp11a	EVM0010707.1	GTGGACGCCTGGGATGGTATCT	ACTCCTCCAGCCATTAGTTCGGTAA	185	103.8%	60
hsd17b12a	EVM0019177.1	CTGGAGGTCGGAGTTCTGGTGAA	TTGGAGGCGGAGTAGACAGTGAG	245	99.3%	60
TGFβ-2	EVM0020611.1	CGCCAACAGCCTGAACCACAA	ACTGCTTCTCCACCTTCCTCTCC	263	101.6%	60
foxo3	EVM0006504.1	TGTCGTGACCAGTAGTGATTGG	TCCAGTTGATACTCACACTGGC	229	98.0%	60
hipk1	EVM0014492.1	TTGGATGGACGCACGGAGACA	ACCTCTGGTGCTCTGTAGCCTATG	163	95.5%	60
hipk2	EVM0017866.1	ATGCTGGCTACGATGTGCTGAAC	GGCTGACCTCTGGTTGTCTGGATA	118	97.4%	60
hipk3	EVM0014492.1	TGGAGACTGAAGACGAAGGAGGA	GCAGCATACGCTTGAGGAGACTC	108	95.8%	60
mapk14a	EVM0020655.1	GCCAGACAGACGGAGAGTGAGAT	GGAGGAAGACCCAACACGAATGAC	278	97.1%	60
gmpr	EVM0000314.1	TGGAGGTGAAGGTGGTACGATGAG	TGTTGGTCTTGGCTGCTGGATTATC	242	94.2%	60
xdh	EVM0005591.1	AACGGCAAGAAGGTGGTGGAGA	TGTAAGGAGCAGAGCGGAGTGAG	212	96.4%	60
hprt1	EVM0006830.1	AGGAGCCAATGGTGGATCTGGAATA	AGTCTCGGAGCAGTCGGTCAAG	219	106.8%	60
nme2	EVM0014703.1	GCAGGCTTCTGAGGAGCACTTG	GTTGGTCTCACCGAGCATCATACG	163	96.2%	60
nme4	EVM0015976.1	AGGTGTCCGTCCGCAGATTCTC	TCGTCCATGAGGAAGGCAGAGTAC	142	105.4%	60
ef1α	EVM0020431.1	CCACCTATGGGAGCAGAAAACT	CTTCAGAGCCTTGGGATTGTCT	189	95.7%	60
β-actin	EVM0008407.1	GTACGACCACTCCATGAAGGTT	TGCCAAAGTTGTCGTTGATGAC	92	96.8%	60
gapdh	EVM0008723.2	CATGAAGTGCGATGTCGACATC	GGCCAGACTCATCATACTCCTG	254	98.6%	60
