# Supplementary material for: Transcriptomics Analysis of Testis Development in Thamnaconus septentrionalis Responding to a Rise in Temperature
Source: Animals (Basel). 2026 Jan 21;16(2):327. doi: 10.3390/ani16020327 (PMC12837544; doi:10.3390/ani16020327)
Supplement: Supplementary file 1 [file animals-16-00327-s001.zip › animals-4077258-supplementary/Table S2.pdf]

Table S2 The statistics of the transcriptomic sequencing data in this study

Sample	Raw reads	Raw bases	Clean reads	Clean bases	Mapping rate (%)	Q20	Q30	GC%
LT1	47922572	7.19G	46378892	6.96G	78.07	96.18	90.66	52.50
LT2	45914410	6.89G	44141932	6.62G	78.36	96.25	90.84	52.19
LT3	46138880	6.92G	43792912	6.57G	78.00	96.68	91.77	51.82
LT	46658621	7.00G	44771245	6.72G	78.14	96.37	91.09	52.17
HT1	43539892	6.53G	41922398	6.29G	78.27	96.32	90.91	52.43
HT2	47225020	7.08G	45442716	6.82G	77.51	96.34	91.00	52.00
HT3	42255874	6.34G	40886606	6.13G	79.03	96.34	91.02	52.61
HT	44340262	6.65G	42750573	6.41G	78.27	96.33	90.98	52.35
