# Supplementary figures and images for: Transcriptomics Analysis of Testis Development in Thamnaconus septentrionalis Responding to a Rise in Temperature
Source: Animals (Basel). 2026 Jan 21;16(2):327. doi: 10.3390/ani16020327 (PMC12837544; doi:10.3390/ani16020327)

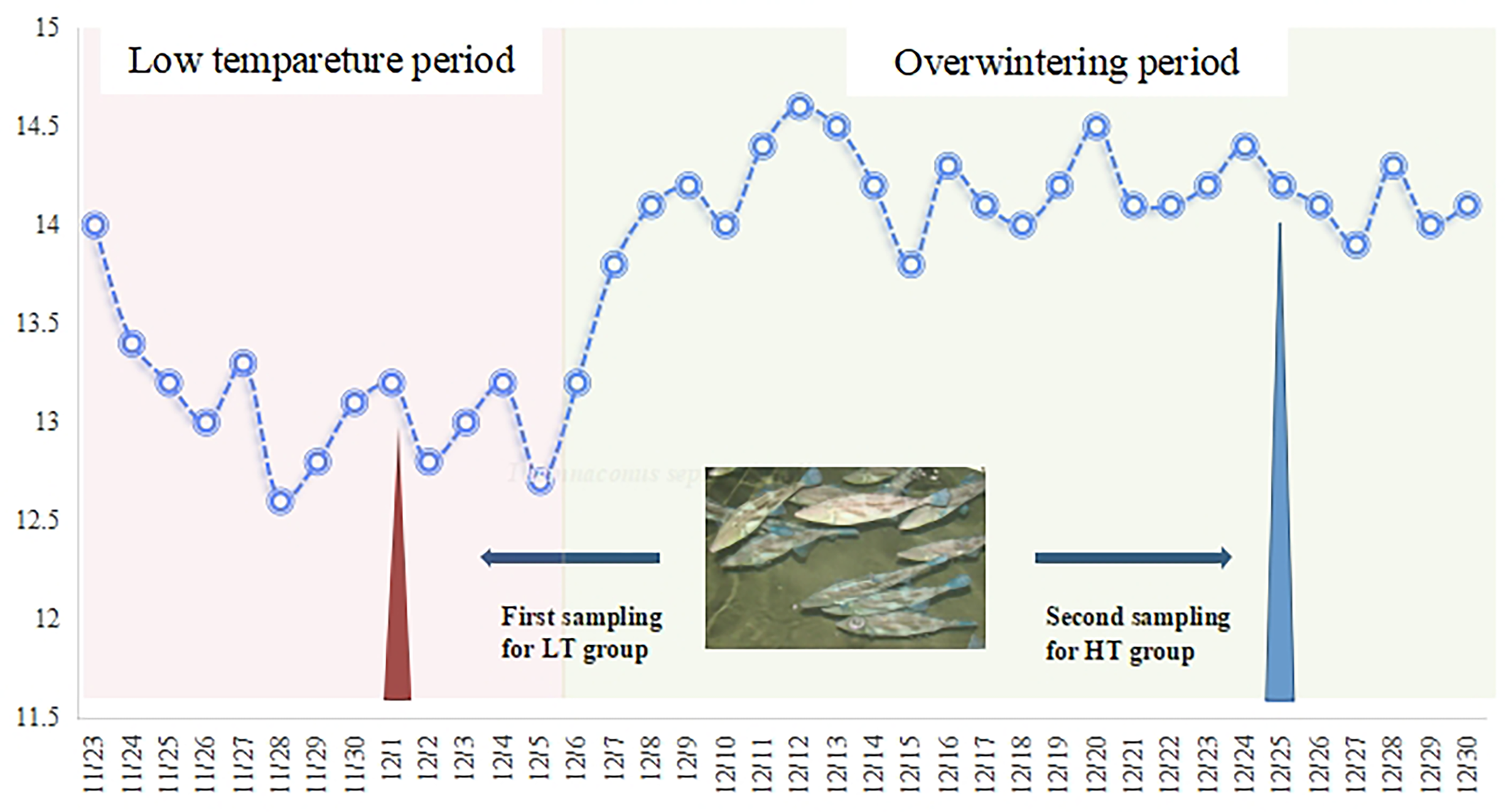

Supplement: Supplementary file 1 [file animals-16-00327-s001.zip › animals-4077258-supplementary/Fig. S1.tif]
